# In-depth biological characterization of two black soldier fly anti-*Pseudomonas* peptides reveals LPS-binding and immunomodulating effects

**DOI:** 10.1128/msphere.00454-23

**Published:** 2023-10-06

**Authors:** Laurence Van Moll, Milan Wouters, Jeroen De Smet, Linda De Vooght, Peter Delputte, Mik Van Der Borght, Paul Cos

**Affiliations:** 1 Laboratory of Microbiology, Parasitology and Hygiene (LMPH), Faculty of Pharmaceutical, Biomedical and Veterinary Sciences, University of Antwerp, Antwerp, Belgium; 2 Department of Microbial and Molecular Systems (M2S), Research Group for Insect Production and Processing (IP&P), KU Leuven, Campus Geel, Geel, Belgium; University of Maryland Medical Center, Baltimore, Maryland, USA

**Keywords:** *Pseudomonas aeruginosa*, antimicrobial peptides, LPS binding, black soldier fly, lipopolysaccharide

## Abstract

As effector molecules of the innate immune system, antimicrobial peptides (AMPs) have gathered substantial interest as a potential future generation of antibiotics. Here, we demonstrate the anti-*Pseudomonas* activity and lipopolysaccharide (LPS)-binding ability of HC1 and HC10, two cecropin peptides from the black soldier fly (*Hermetia Illucens*). Both peptides are active against a wide range of *Pseudomonas aeruginosa* strains, including drug-resistant clinical isolates. Moreover, HC1 and HC10 can bind to lipid A, the toxic center of LPS and reduce the LPS-induced nitric oxide and cytokine production in murine macrophage cells. This suggests that the peptide-LPS binding can also lower the strong inflammatory response associated with *P. aeruginosa* infections. As the activity of AMPs is often influenced by the presence of salts, we studied the LPS-binding activity of HC1 and HC10 in physiological salt concentrations, revealing a strong decrease in activity. Our research confirmed the early potential of HC1 and HC10 as starting points for anti-*Pseudomonas* drugs, as well as the need for structural or formulation optimization before further preclinical development can be considered.

**IMPORTANCE:**

The high mortality and morbidity associated with *Pseudomonas aeruginosa* infections remain an ongoing challenge in clinical practice that requires urgent action. *P. aeruginosa* mostly infects immunocompromised individuals, and its prevalence is especially high in urgent care hospital settings. Lipopolysaccharides (LPSs) are outer membrane structures that are responsible for inducing the innate immune cascade upon infection. *P. aeruginosa* LPS can cause local excessive inflammation, or spread systemically throughout the body, leading to multi-organ failure and septic shock. As antimicrobial resistance rates in *P. aeruginosa* infections are rising, the research and development of new antimicrobial agents remain indispensable. Especially, antimicrobials that can both kill the bacteria themselves and neutralize their toxins are of great interest in *P. aeruginosa* research to develop as the next generation of drugs.

## INTRODUCTION

As cases of antibiotic resistance continue to rise exponentially, the development of new antibacterial agents that retain activity against difficult-to-treat bacteria remains indispensable (1). *Pseudomonas aeruginosa* is part of the ESKAPE pathogens (*Enterococcus faecium*, *Staphylococcus aureus*, *Klebsiella pneumoniae*, *Acinetobacter baumannii*, *Pseudomonas aeruginosa*, and *Enterobacter* spp.), a group of bacteria that pose a considerable threat to public health due to their increasing acquisition of multi-drug resistance genes and their wide distribution in both community and healthcare settings (2). Moreover, in 2017, *P. aeruginosa* was listed by the World Health Organization as a critical priority pathogen for which new antibiotics are urgently needed (3). The emergence of carbapenem-resistant *P. aeruginosa* specifically severely limits the treatment options for multi-drug-resistant infections (2).

*P. aeruginosa* is a Gram-negative, aerobic, rod-shaped bacterium that has the ability to survive in a multitude of natural habitats, including water and soil (4 – 6). As an opportunistic pathogen, it mostly causes infection in immunocompromised individuals, including cystic fibrosis, cancer, and burn wound patients (7). *P. aeruginosa* is also a frequent cause of nosocomial infections, manifesting, for example, as surgical site or bladder infections, sepsis, and ventilator-associated pneumonia, for instance, in COVID-19 patients (8). Apart from the bacterium’s acquired resistance, the high mortality rates seen in hospitalized patients are partially due to unique intrinsic and adaptive resistance mechanisms, which include biofilm and persister cell formation (9, 10). *P. aeruginosa* secretes a remarkably diverse array of virulence factors, contributing to the bacterium’s ability to adapt to challenging conditions such as the host’s immune response (7, 11). To counteract these difficult-to-treat infections, there is increasing interest in new, alternative therapeutic agents, such as antimicrobial peptides (AMPs) (12).

AMPs, or host-defense peptides, are small, amphipathic, mostly cationic peptides transcribed in all kingdoms of life, from prokaryotes to humans (13). As effector molecules of the innate immune response, they often have potent activity against a range of different microorganisms (14). Moreover, the fast bacterial killing of AMPs by binding non-protein targets, including bacterial membranes, slows the development of antimicrobial resistance (15 – 17). Many AMPs also have a well-documented immunomodulating activity (18). By regulating gene expression in immune cells, modulating chemotaxis of leukocytes, activating the complement system, and stimulating angiogenesis, AMPs are able to directly influence the course of the immune response (13, 18, 19). Some AMPs, including the human cathelicidin LL-37, are also able to bind and neutralize lipopolysaccharide (LPS), an outer membrane structure of Gram-negative bacteria (19 – 21). LPS, also called endotoxin, will activate a cascade of immune reactions by binding toll-like receptor 4 after being released into the bloodstream, which can ultimately lead to sepsis (20). The combination of certain AMPs’ potent and rapid antimicrobial activity, lower propensity toward resistance development, and their anti-inflammatory action by binding LPS makes them interesting starting points for anti-*Pseudomonas* drugs (18). However, the development of AMPs into successful antibiotics has so far been hampered by some intrinsic peptide limitations, such as poor metabolic stability, low bioavailability, and decreased activity in physiological conditions due to salt interactions (22, 23). Moreover, many AMPs show some degree of unselective cell binding, leading to unwanted side effects such as hemolysis (24).

In previous research, we performed a broad antimicrobial screening of a collection of black soldier fly AMPs (25). The black soldier fly (*Hermetia Illucens*) is a saprophytic, true fly (Diptera), originating from the neotropical region of central and southern America (26, 27). It possesses the second largest repertoire of AMPs ever recorded in insects (26, 28). Strong antibacterial activity in the Gram-negative spectrum was found for its cecropin AMPs, a subfamily of alpha-helical peptides, including high anti-*Pseudomonas* activity. Two cecropins, HC1 and HC10, were selected for detailed *in vitro* characterization (25, 29). We showed that HC1 and HC10 are both highly active, low-hemolytic peptides with membrane permeabilizing effects (25). The ability to bind LPS and maintain antimicrobial activity at physiological salt concentration are important factors when considering developing AMPs into treatment agents. Therefore, in this paper, we further characterize the *in vitro* activity profile of HC1 and HC10, with a focus on their salt sensitivity and LPS-neutralizing effects.

## MATERIALS AND METHODS

### Materials and peptide synthesis

HC1 and HC10 were synthesized using conventional solid-phase peptide synthesis using a 9-fluorenylmethoxycarbonyl protection strategy by Proteogenix (France). Trifluoroacetic acid was used as a deprotection agent. Both peptides were purified using reverse-phase liquid chromatography with a purity of >90%. Data of peptide characterization including high-performance liquid chromatography (HPLC) and mass spectrometry were enclosed by the manufacturer. HC1 is a 44-amino acid-long peptide, while HC10 has 47 amino acids. Both peptides are amidated at the C-terminus (25). For the experiments, AMP stock solutions of 10 mM were prepared in dimethyl sulfoxide (Acros Organics) and further diluted in sterile demineralized water. The LPS from *P. aeruginosa* used in the experiments was purified by phenol extraction and bought from Sigma-Aldrich.

### Cell culture and viability

The murine macrophage cell line RAW264.7 (ATCC TIB-71^M^) was cultivated in Dulbecco’s modified eagle medium (DMEM), supplemented with 10% heat-inactivated fetal bovine serum and 2 mM L-glutamine, at 37°C and 5% CO_2_. For the cell viability assay, HC1 and HC10 (32–2 µM) with or without the addition of LPS (100 ng/mL), LPS alone (100 ng/mL), and tamoxifen (128–1 µM, Sigma) were prepared in DMEM and added to 96-well plates. Tamoxifen was included as a cytotoxic reference compound. As a negative control, wells with untreated cells were included. Next, RAW264.7 cell suspension was added to the plates at a concentration of 1.5 × 10^5^ cells/mL. After 48 h of incubation, 50 µL of a 0.01% resazurin solution (Sigma-Aldrich) was added to each well. Resazurin is a commonly used redox indicator that undergoes a fluorescent color change in the presence of viable cells (30). After 4 h, the fluorescent signal was read using a plate reader (GloMax, Promega) at *λ*_excitation_= 550 nm and *λ*_emission_= 590 nm. The fluorescent data were used to calculate the cell viability percentage. The viability assay was performed in biological triplicate.

### Antimicrobial activity screening

The activity of HC1 and HC10 against a broad range of clinical and non-clinical *P. aeruginosa* isolates was investigated using an antimicrobial screening assay with resazurin as a viability indicator. The panel consisted of *P. aeruginosa* PAO1, *P. aeruginosa* PA14, *P. aeruginosa* LMG 27650, *P. aeruginosa* AA2, *P. aeruginosa* RP73, *P. aeruginosa* NH57388A, *P. aeruginosa* AMT0023-34, *P. aeruginosa* LMG 14084, and *P. aeruginosa* PR355. For the screening, serial dilutions of the peptides were prepared in technical triplicate in Müeller Hinton broth (MHB, Difco) in 96-well plates in a final volume of 100 µL. Test concentrations started at 32 µM. Polymyxin B, a clinically used peptide antibiotic with a Gram-negative spectrum, was included as a reference compound (31). Afterwards, 100 µL of bacterial *P. aeruginosa* suspension was added to the test plates, to a final inoculum concentration of 5 × 10^4^ CFU/mL. Plates were incubated overnight at 37°C. The next day, 20 µL of a 0.01% resazurin solution was added to each well. After the reduction of resazurin to resorufin (4 h for *P. aeruginosa* RP73 and NH57388A, 15 min for all other isolates), the fluorescent signal was read using a plate reader (Glomax, Promega) at *λ*_excitation_= 550 nm and *λ*_emission_= 590 nm. The fluorescent data were used to calculate the concentration of the peptides leading to 50% growth inhibition of the bacteria (IC50). The minimum inhibitory concentration (MIC) was determined visually as the lowest concentration where no bacterial growth occurred, according to the EUCAST (2022) guidelines (32). At least two biological repeats of the screening assay were performed. The IC50 values are reported as the mean ± standard deviations of all technical and biological repeats. For the MIC values, a concentration range was reported, as these concentrations represent discrete instead of continuous values.

### Activity of HC1 and HC10 in simulated lung medium

To test the activity of HC1 and HC10 in simulated lung conditions, an antimicrobial screening experiment was carried out in a modified Gamble’s solution with resazurin as a viability indicator. Briefly, the Gamble’s solution was prepared as described by Calas et al., but components were dissolved in Müeller Hinton bacterial medium (pH 7.4) (33). The antimicrobial activity against *P. aeruginosa* PAO1 was determined similar to the protocol described earlier in the text. The fold increase in activity (IC50 value) compared to non-supplemented bacterial broth was calculated. This experiment was performed in biological triplicate.

### Salt sensitivity of HC1 and HC10

To test the sensitivity of the AMPs to salts, a resazurin-based antimicrobial screening experiment against *P. aeruginosa* PAO1 was performed in the presence of increasing concentrations of NaCl and CaCl_2_. Briefly, the MHB was supplemented with either 10, 25, 50, 75, 100, 150, or 300 mM of NaCl and 0.05, 0.1, 0.25, 0.5, 1, or 2.5 mM of CaCl_2_. The antimicrobial activity was determined similar to the protocol earlier described in the text. The activity in salt-supplemented conditions was compared to the activity in the normal, non-supplemented medium control by calculating the fold change in IC50 value. This experiment was carried out in biological triplicate.

### Endotoxin neutralization using the limulus amebocyte lysate assay

LPS neutralization was investigated using a commercially available chromogenic limulus amebocyte (LAL) assay (Toxinsensor, Genscript). Peptides were prepared in endotoxin-free water at final concentrations ranging from 32 to 1 µM and mixed with 0.5 endotoxin units/mL of standard *E. coli* endotoxin. After 30 min of incubation at 37°C, the amount of free endotoxin was determined according to the manufacturer’s instructions. Absorbance was read at 560 nm on a plate reader (GloMax, Promega). A standard curve was included to calculate the amount of free endotoxins present in the samples. The LAL assay was performed in biological triplicate.

### LPS binding using BODIPY TR cadaverine displacement assay

To determine the binding of HC1 and HC10 to the lipid A part of LPS, a BODIPY TR cadaverine (BC, Invitrogen) displacement assay was carried out. Serial dilutions of HC1 and HC10 (32–2 µM) were prepared in technical triplicate in 50 mM Tris buffer (pH 7.4) in black 96-well plates. LPS and BC solutions in Tris buffer were added at final concentrations of 100 ng/mL and 2.5 µM, respectively. Fluorescence was read using a plate reader (Infinite F Plex, Tecan) at *λ*_excitation_= 580 nm and *λ*_emission_= 620 nm during a 1-h cycle. Additionally, the effect of divalent cations on the AMP-LPS binding was studied by repeating the assay in 2.5 mM CaCl_2_-supplemented Tris buffer. Both BC displacement assays were performed in biological triplicate.

### Nitrite detection using the Griess reaction in LPS stimulated RAW264.7 cells

To determine the amount of nitrite produced by RAW264.7 macrophages after LPS and/or AMP treatment, a Griess reaction (Invitrogen) was performed. Cells were seeded in 96-well plates at a density of 5 × 10^5^ cells/mL. After 24 h of incubation, the cell medium was discarded and replaced by AMP serial dilutions in DMEM in technical duplicate (32–2 µM) with or without the addition of LPS (100 ng/mL). Controls of LPS without peptide (100 ng/mL) and DMEM medium were included in the test as well. After 48 h of incubation, the nitrite content in the cell supernatant was determined following the kit manufacturer’s instructions. Briefly, 20 µL of a mixture of equal volumes of N-(1-naphthyl)ethylenediamine and sulfanilic acid were added to 150 µL of cell supernatant. After 30 min, the absorbance was read at 560 nm using a plate reader (GloMax, Promega). A nitrite standard curve was included to calculate the amount of nitrite present in the supernatant. The Griess reaction was performed in biological triplicate.

### Detection of TNF-α and IL-6 in LPS-stimulated macrophages using enzyme-linked immunosorbent assays

Enzyme-linked immunosorbent assays (ELISA, Invitrogen) were used to determine the amount of TNF-α and IL-6 present in the supernatant of LPS- and/or AMP-treated RAW264.7 cells. Macrophages were seeded and treated similarly as described for the nitrite detection. After 24 h of treatment, the TNF-α and IL-6 levels were determined following the manufacturer’s instructions. After the addition of the stop solution (1 M phosphoric acid), the absorbance of the microwells was read using a plate reader (GloMax, Promega) at 450 nm. TNF-α and IL-6 levels were calculated using the absorbance data of the standard curves. The ELISA experiments were carried out in biological triplicate.

### Detection of TNF-α, IL-1β, IL-6, and IL-12β in LPS-stimulated macrophages using qPCR

RAW264.7 cells were seeded in 24-well plates at a density of 5 × 10^5^ cells/mL and treated similarly as described earlier for the nitrite detection. After 4 h of LPS and/or peptide treatment, total RNA was extracted from the cells using TRIzol reagent (Ambion), according to the manufacturer’s instructions. The resulting RNA was treated with ezDNAse (Invitrogen), and cDNA was prepared using the Superscript IV reverse transcriptase (Invitrogen). The SensiFAST SYBR No-ROX One-Step Kit (Bioline) was used to amplify the genes of interest from the cDNA. Mouse-specific primers for TNF-α (forward: CATCTTCTCAAAATTCGAGTGACAA, reverse: TGGGAGTAGACAAGGTACAACCC), IL-1β (forward: CAACCAACAAGTGATATTCTCCATG, reverse: GATCCACACTCTCCAGCTGCA), IL-6 (forward: GAGGATACCAC-TCCCAACAGACC, reverse: AAGTGCATCATCGTTGTTCATACA), and IL-12β (forward: GGAAGCACGGCAGCAGAATA, reverse: AACTTGAGGGAGAAGTAGGAATGG) were used. GAPDH (forward: TCACCACCATGGAGAAGGC, reverse: GCTAAGCAGTTGGTGGTGCA) was used as the housekeeping gene control. The Lightcycler 480 II System and corresponding software (Roche Diagnostics) were used to perform the qPCR experiments. The polymerase was activated at 95°C for 2 min, followed by 40 cycles of two-step amplification (denaturation at 95°C for 5 s, followed by an annealing step at 60°C for 20 s). Relative gene expression, normalized to GAPDH, was calculated using the Pfaffl method (34). Three biological repeats of the qPCR experiments were carried out.

### Statistical analysis

Results are reported as mean ± standard deviation of all technical and biological replicates. For the salt sensitivity assay, a one sample *t*-test was used to determine if the fold increase in IC50 was significantly different from 1. For the BC displacement assay, a one-way ANOVA test was used to determine the significant difference between groups after 1 h of treatment. As not all data sets were normally distributed, but most had equal variances for the ELISA and qPCR experiments, a non-parametric Kruskal-Wallis test was used to compare the treated samples with the controls. Graphpad Prism 9.5 was used for all data visualization and statistical analysis.

## RESULTS

### Antimicrobial activity of HC1 and HC10 against a panel of *P. aeruginosa* isolates

The activity of HC1 and HC10 against a panel of eight different *P. aeruginosa* isolates, chosen to represent a broad variety in origin (clinical or environmental) and phenotype, was investigated (35 – 37). Both peptides showed activity against all strains tested, including intermediate and multi-drug-resistant strains. IC50 values for all isolates are in the low micromolar range (Table 1). Activity of the clinically used polymyxin B is higher than that of HC1 and HC10 but in the same concentration range. *P. aeruginosa* PA14, a highly virulent strain causing acute infections, was noticeably more susceptible to both HC1 and HC10 and polymyxin B (38). In contrast, HC1, HC10, and polymyxin B were less active against the low-inflammatory *P. aeruginosa* RP73 isolate from a patient with cystic fibrosis, showing a threefold increase in IC50 value compared to PAO1 (36).

**TABLE 1:** Antimicrobial activity of HC1 and HC10 against a panel of *P. aeruginosa* isolates^*a*^

Strain	Origin	Resistant?	Average IC50 (µM)			Range MIC (µM)	
			HC1	HC10	P^*d*^	HC1	HC10
PAO1	Non-CF^*b*^, wound (39)	No	1.38 ± 0.37	1.15 ± 0.36	0.53 ± 0.15	2	2
PA14	Non-CF, burn wound ( 38, 39)	No	0.47 ± 0.16	0.63 ± 0.17	0.16 ± 0.04	1–2	1–2
LMG 27650	Non-CF, clinical (40)	Yes (MDR^*c*^)	1.39 ± 0.37	0.99 ± 0.40	0.42 ± 0.18	2–4	2
AA2	CF (acute) (41)	Cefoperazone, piperacillin	1.31 ± 0.35	1.11 ± 0.31	0.51 ± 0.25	2	2
RP73	CF (chronic) (36)	Meropenem, ceftazidime, gentamicin	3.91 ± 1.27	3.73 ± 1.47	1.00 ± 0.22	4–8	4–8
NH573-88A	CF (chronic) (37)	Quinolones, β-lactam antibiotics	1.39 ± 0.18	1.28 ± 0.12	0.30 ± 0.06	2	2
AMT0023-34	CF (acute) (37)	Quinolones, amino-glycosides	0.89 ± 0.63	0.95 ± 0.37	0.37 ± 0.05	1–2	1–2
LMG 14084	Non-clinical, environment (40)	No	1.48 ± 0.26	1.00 ± 0.47	0.52 ± 0.06	2	2
PR355	Non-clinical, hospital (40)	No	1.19 ± 0.43	1.17 ± 0.46	0.35 ± 0.09	1–2	1–2

^*a*^ IC50 values (concentration of peptide leading to 50% of growth inhibition) and the MIC value in the low micromolar range show the high activity of HC1 and HC10 against all strains tested. Polymyxin B was included as a reference antibiotic. Data are represented as the mean ± the standard deviation of all technical and biological repeats.

^*b*^ CF, cystic fibrosis.

^*c*^ MDR, multi-drug resistant.

^*d*^ P, polymyxin B.

### Salt sensitivity of HC1 and HC10

**Fig 1:**
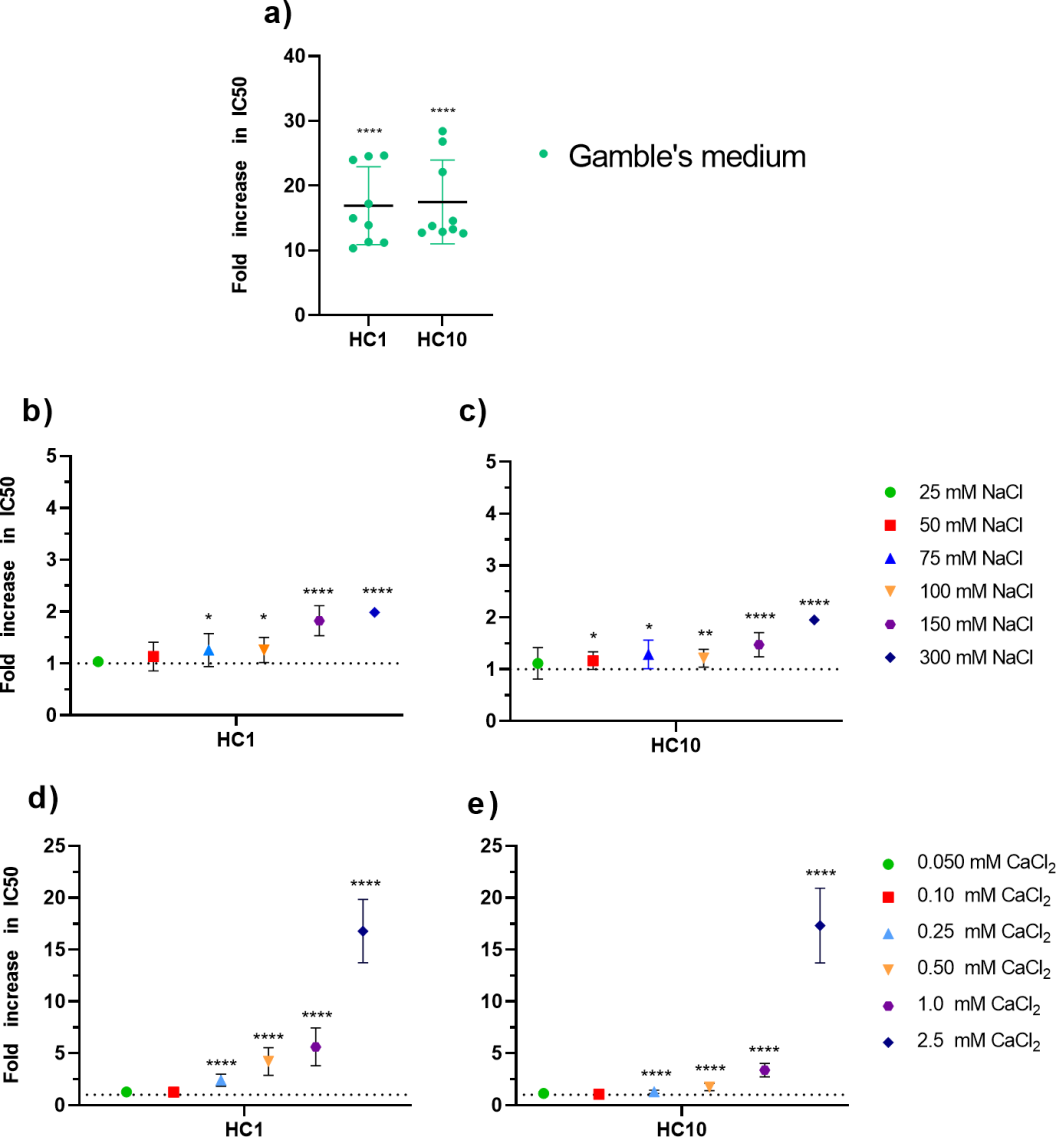
Salt sensitivity of HC1 and HC10. (**a**) The activity of HC1 and HC10 against *P. aeruginosa* PAO1 strongly decreases in simulated lung fluid conditions (Gamble’s medium). (**b and c**) The anti-*Pseudomonas* activity of HC1 and HC10 slightly decreases in the presence of high (≥75 mM) concentrations of NaCl. (**d and e**) The addition of CaCl_2_ has a clear, negative impact on the antimicrobial activity of HC1 and HC10. At physiological concentrations (2.5 mM), the activity strongly decreased. Data are presented as mean ± standard deviation. Data were analyzed using a one sample *t*-test to test for significant difference from 1. *P ≤ 0.05, **P ≤ 0.01, ***P ≤ 0.001, and ****P ≤ 0.0001. All experiments were carried out in biological triplicate.

As *P. aeruginosa* is a pathogen that commonly affects the lungs, HC1 and HC10 were screened in a simulated lung fluid medium (Gamble’s solution). Lung fluid is rich in various salts, which are known to interact with AMPs (42). Both HC1 and HC10 indeed showed a strong increase in IC50 values, indicating a decreased anti-*Pseudomonas* activity in the Gamble’s medium (Fig. 1a). To study whether the decrease in antimicrobial activity of HC1 and HC10 occurred due to the presence of monovalent or divalent salts, the peptides were screened against *P. aeruginosa* in increasing concentrations of NaCl and CaCl_2_. The addition of NaCl did not have a significant effect on the antimicrobial activity of HC1 and HC10 up until the concentrations of 75 mM (Fig. 1b and c). At 100 mM NaCl, the concentration of salt present in lung fluid, the activity only minorly decreased compared to the activity in a non-supplemented medium, with a 1.3-fold increase in IC50 for HC1 and a 1.2-fold increase for HC10 (33). In 150 mM NaCl, approximately the concentration found in human blood, the IC50 increased by a factor of 1.8 for HC1 and 1.5 for HC10 (43). The effect of CaCl_2_ on the AMP antimicrobial activity was more outspoken than that of NaCl. For both peptides, a significant decrease in activity was found from 0.25 mM CaCl_2_ onwards (Fig. 1d and e). At 2.5 mM CaCl_2_, the antimicrobial activity strongly decreased, with an average 17-fold increase in IC50 for both HC1 and HC10. As this is also the concentration found in human lung fluid, the presence of divalent salts presumably causes a significant decrease in activity in lung conditions (33).

### LPS neutralization by HC1 and HC10

**Fig 2:**
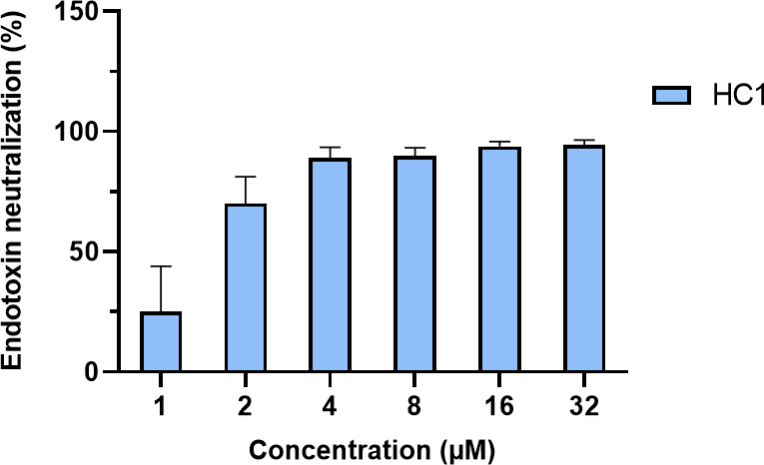
LPS neutralization by HC1, as determined by a chromogenic LAL assay. HC1 shows a concentration-dependent neutralization of standard *E. coli* endotoxin, with high neutralization from 4 µM onwards. Data for HC10 are not included in the graph, as this peptide intrinsically induced the LAL enzyme cascade, leading to high background signals. The experiment was carried out in biological triplicate. Data are represented as mean + standard deviation of all technical and biological replicates.

The chromogenic LAL assay was used to study the ability of HC1 and HC10 to neutralize free-circulating, standard *E. coli* endotoxin. The assay makes use of an extract of blood cells from the Horseshoe Crab, which contains a pro-enzyme that is activated in the presence of endotoxins or LPS (44, 45). After a catalytic cascade of reactions, the colored product *p*-nitroaniline is released, which can be quantified spectrophotometrically (44). HC1 was able to neutralize the added endotoxin in a concentration-dependent manner. At concentrations as low as 4 µM, 89% LPS neutralization was observed (Fig. 2). LPS neutralization for HC10, however, could not accurately be calculated and is, therefore, not included in the graph below. Upon addition of HC10, a high background signal was obtained, even in endotoxin-free peptide controls, indicating that HC10 autonomously induces the LAL pro-enzyme.

### Binding of HC1 and HC10 to lipid A and effect of CaCl_2_

**Fig 3:**
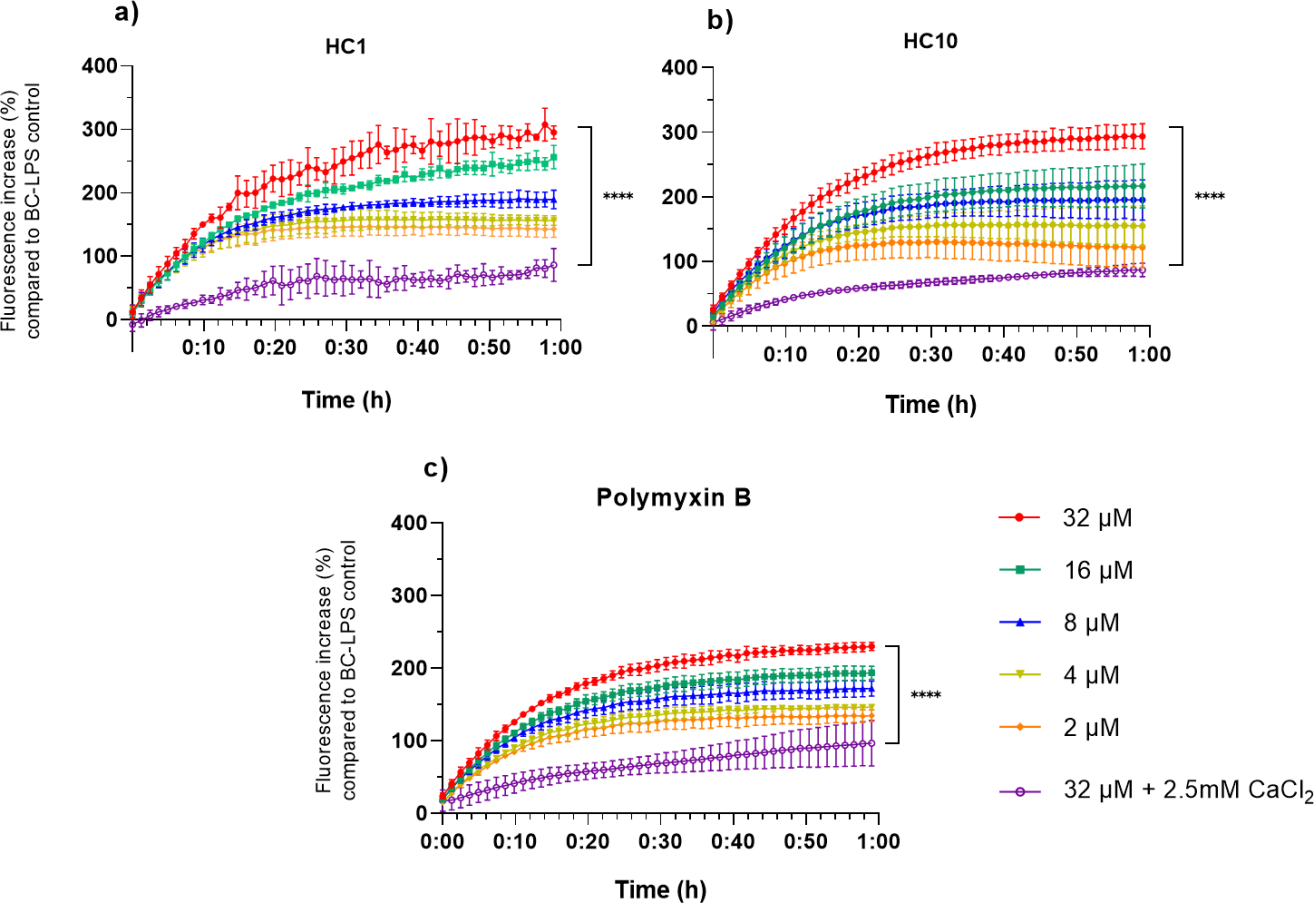
BODIPY-TR cadaverine displacement assay. (**a and b**) HC1 and HC10 are able to displace BC in a concentration-dependent manner, signified by the increase in fluorescence over time. As a control, samples with BC and LPS (100 ng/mL) without peptide treatment were included. At 32 µM of AMP, there is close to a 300% increase in fluorescence compared to the control. (**c**) BC displacement assay for the peptide antibiotic reference polymyxin B. The LPS binding of polymyxin B follows a similar concentration-dependent trend over time, but for 16 and 32 µM, the polymyxin-induced LPS binding is lower than for HC1 and HC10. Fluorescence was read during a 1-hour cycle. Data are represented as mean ± standard deviation. Data sets at the last timepoint were compared with a one-way ANOVA test, ****P ≤ 0.0001. The assay was carried out in biological triplicate.

Lipid A is the pyrogenic part of LPS, responsible for triggering the innate immune response via toll-like receptor 4 (46). Structurally, it is the most conserved part of LPS, consisting of a hydrophilic amino disaccharide backbone and a hydrophobic domain of fatty acids tails (46, 47). To study the affinity of the AMPs to the lipid A toxic center of LPS, a fluorescent displacement assay using BC was carried out (48). BC, cadaverine linked to the fluorescent dye BODIPY TR, will bind to lipid A via electrostatic interactions, which decreases its fluorescent signal. When BC is displaced from its interaction with lipid A due to competition with other LPS-binding compounds, dequenching of its fluorescence will occur (47, 48). Both AMPs show a concentration-dependent increase in fluorescence, compared to the non-peptide treated control (Fig. 3a and b). Displacement of BC by the AMPs occurs fast and plateaus after 20 min of treatment. At MIC concentrations (2 µM), there is already a high amount of AMP-LPS binding, signified by the over 100% increase in fluorescence. At 32 µM, there is a 295% increase in fluorescence for HC1 and a 294% increase for HC10. For polymyxin B (Fig. 3c), the LPS-binding trend is similar; however, the increase in fluorescence at 32 µM only reaches approximately 220%, significantly lower (*P* ≤ 0.5) than for HC1 and HC10. To test the effect of divalent cations on the lipid A binding, the assay was repeated in 2.5 mM CaCl_2_-supplemented buffer. In the presence of divalent salts, the LPS binding is noticeably lower for HC1, HC10, and polymyxin B, as the fluorescence increased only marginally at high concentrations of 32 µM.

### Inhibition of LPS-induced macrophage activation

**Fig 4:**
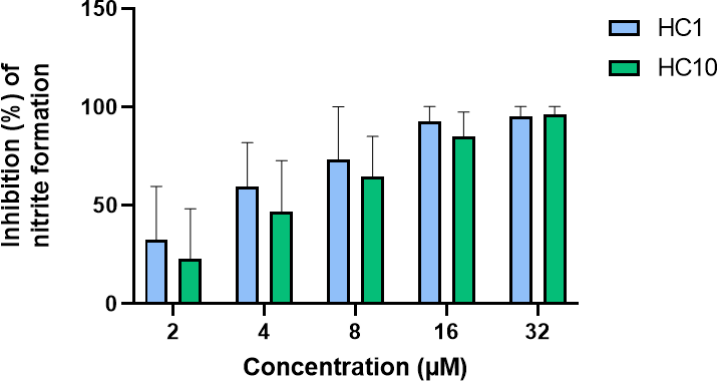
Effect of HC1 and HC10 on nitrite production by LPS-stimulated RAW264.7 macrophages as determined by a Griess reaction. HC1 and HC10 have a concentration-dependent effect on the macrophage activation after LPS addition, presumably through their LPS-binding ability. Both peptides achieve high, near 100% inhibition of nitrite formation at concentrations of 32 µM. As a positive control, LPS-stimulated macrophages without peptides were used. The experiment was carried out in biological triplicate. Data are represented as mean + standard deviation.

To determine whether the LPS-AMP binding also decreases macrophage activation, a Griess reaction was performed. When macrophages are activated upon contact with LPS, they release the unstable nitric oxide, which is quickly converted into the more stable nitrite (49). Hence, nitrite, which can be detected by the chromogenic Griess reaction, is a useful parameter for macrophage activation. HC1 and HC10 showed a concentration-dependent effect on the nitrite release by LPS-stimulated murine macrophages (Fig. 4). At lower concentrations, the inhibition of nitrite production was more variable than at higher concentrations (Fig. 4). However, at 32 µM, both peptides consistently achieved close to 100% inhibition of nitrite formation. To ensure that any observed effects were not due to decreased cell viability, the effect of the AMPs, either in monotreatment or in co-treatment with LPS, and LPS (100 ng/mL), on RAW264.7 cell viability was checked with a resazurin assay. No significant change in viability was observed (Fig. S1).

### Inhibition of pro-inflammatory cytokine release

**Fig 5:**
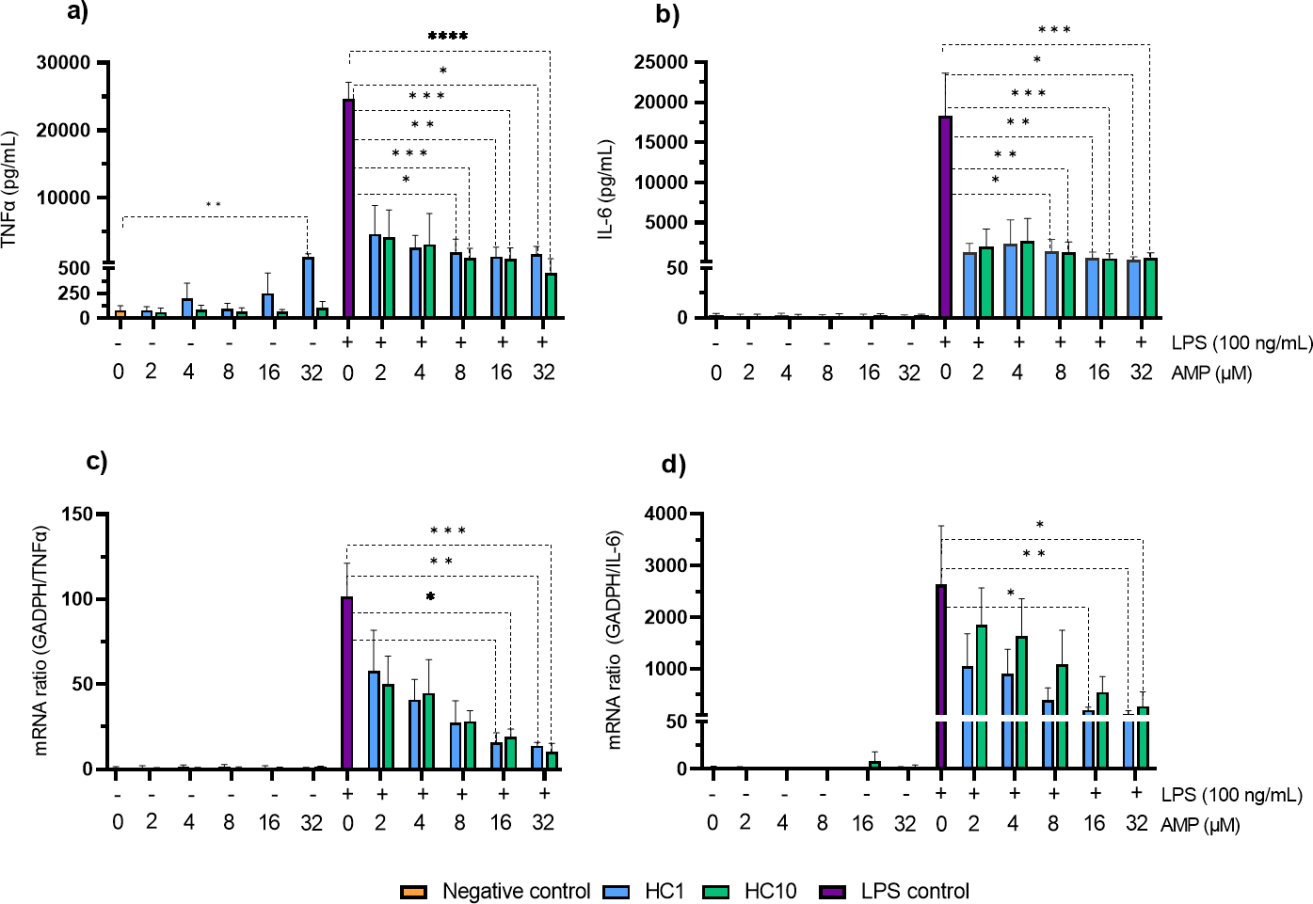
Effect of HC1 and HC10 on the release of the pro-inflammatory cytokines TNF-α and IL-6. (**a**) The release of TNF-α by RAW264.7 macrophages after peptide monotreatment or LPS-AMP co-treatment for 24 h was investigated using ELISA. (**b**) The release of IL-6 by RAW264.7 macrophages after peptide monotreatment or LPS-AMP co-treatment for 24 h was investigated using ELISA. (**c**) qPCR analysis of the release of TNF-α by RAW264.7 macrophages after peptide monotreatment or LPS-AMP co-treatment for 4 h. (**d**) qPCR analysis of the release of IL-6 by RAW264.7 macrophages after peptide monotreatment or LPS-AMP co-treatment for 4 h. LPS-treated cells (100 ng/mL) were included as positive controls. Data are represented as mean + standard deviation and statistically analyzed using a Kruskal-Wallis test. *P ≤ 0.05, **P ≤ 0.01, ***P ≤ 0.001, ***P ≤ 0.0001. All experiments were carried out in biological triplicate.

The effect of the AMPs on the release of the pro-inflammatory cytokines TNF-α and IL-6 by murine macrophages was investigated using both ELISA and qPCR (Fig. 5). Upon co-treatment with LPS, both HC1 and HC10 downregulate the expression of TNF-α and IL-6 in a concentration-dependent manner, while, at near-MIC concentrations (2–4 µM), the effect on the cytokine release was not statistically significant. The decrease in pro-inflammatory cytokine production was consistently high and significant at 32 and 16 µM. qPCR experiments for IL-1β and IL-12β showed comparable results (Fig. S2). In addition, the ELISA experiment showed that at 32 µM, HC1 also moderately induces the expression of TNF-α in the absence of LPS stimulation, indicating that at high concentrations HC1 has intrinsic immunomodulating effects, unrelated to its LPS-binding activity (Fig. 5a). The same effect was not seen for HC10 or for IL-6 expression. Noticeably, this TNF-α induction by HC1 was not observed in the qPCR experiments. As the ELISA measured cytokine levels after 24 h, while the qPCR experiments analyzed the expression of cytokine mRNA after 4 h of treatment, the time difference in exposure to the peptides could explain this effect.

## DISCUSSION

### AMPs as anti-*Pseudomonas* therapeutics

As an opportunistic pathogen that has the ability to cause invasive, difficult-to-treat infections, *P. aeruginosa* poses a significant health threat to immunocompromised individuals (50, 51). Due to growing resistance rates, the need for new, functional antibacterials is high (7). Most *P. aeruginosa* infections are characterized by a strong inflammatory response, which not only leads to acute tissue damage but is also related to poor disease outcome and the bacterium’s ability to cause chronic, persistent infections (52, 53). It has been opted that therapeutics with immunomodulating effects could positively influence the course of a *P. aeruginosa* infection (52). Due to their wide range of bioactivities, AMPs have gathered substantial interest as potential next-generation antimicrobials (54, 55). Since many AMPs can target both the bacterial colonization and inflammation processes during infection, these new peptide therapeutics seem promising to effectively combat Gram-negative bacteria (54, 55). Despite a surge in AMP research and development, infections with Gram-negative pathogens such as *P. aeruginosa* remain largely untargeted by AMPs in clinical trials (56 – 58).

### LPS binding is involved in HC1 and HC10 activity

Two assays were used to detect LPS-AMP binding in our study: an LAL assay and a BC displacement assay. The LAL assay has been commonly used to study endotoxin-neutralization of AMPs (59 – 61). However, due to high background signals caused by HC10, presumably through intrinsic activation of the LAL proenzyme, the LAL assay gave unreliable results for HC10. We used an additional BC displacement assay to confirm the presence of AMP-LPS interactions, a technique that is used less frequently, yet offers a valid alternative for the LAL assay when studying LPS-AMP binding (47). The BC displacement assay confirmed the binding of HC1 and HC10 to lipid A, the toxic center of LPS. Moreover, despite a lower antimicrobial activity against *P. aeruginosa* compared to polymyxin B, the binding of *P. aeruginosa* LPS was higher at 32 µM for HC1 and HC10.

The importance of this LPS-AMP binding for the AMP activity was further supported by the results of our screening of the two black soldier fly AMPs, HC1 and HC10, against a panel of different *P. aeruginosa* isolates. The susceptibility of *P. aeruginosa* RP73, a chronic cystic fibrosis isolate, to the peptides was noticeably lower (36). *P. aeruginosa* RP73 contains low-inflammatory lipo-oligosaccharide molecules that possess under-acylated lipid A structures. In contrast to some other cystic fibrosis isolates, RP73 does not contain LPS mutations that typically confer increased resistance to AMPs, such as the addition of aminoarabinose to the free phosphate groups of the glucosamine backbone (62, 63). However, the under-acylation of LPS molecules has in the past also been linked to increased AMP and polymyxin resistance in various Gram-negative bacteria (64 – 67). For *P. aeruginosa* specifically, strains with penta-acylated LPS structures are less susceptible to polymyxin B due to decreased hydrophobic interactions (67). Our research suggests that the antibacterial activity of HC1 and HC10 cecropins is also influenced by the acylation pattern of lipid A, although other mutations in *P. aeruginosa* RP73 can play a role in its decreased AMP susceptibility. Nevertheless, as *P. aeruginosa* isolates often acquire LPS mutations during chronic infections, screening of AMPs against different clinical isolates ranging from various stages of infection, should not be overlooked during pre-clinical *in vitro* examination of anti-*Pseudomonas* peptides.

### LPS binding decreases inflammatory responses

To verify whether LPS binding by AMP also had an effect on the inflammatory response caused by LPS, a series of experiments were performed. The release of various pro-inflammatory mediators, including nitric oxide, IL-6, and TNF-α, by murine macrophages after AMP and LPS co-treatment was investigated. Both HC1 and HC10 reduced the inflammatory effects caused by LPS, indicating that the AMP-LPS complex is less prone to triggering the immune cascade. Similar anti-inflammatory effects through LPS binding have been described for other cecropin AMPs as well, including Papiliocin and SibaCec (68, 69). The underlying mechanism is thought to involve a change in the aggregate structure of free LPS by AMP interaction. LPS-binding AMPs are either able to break up the LPS micelles in solution, creating smaller, unorganized particles, or increase LPS aggregation, forming large multilamellar structures. In both cases, LPS becomes less available for LPS-binding protein, which in turn leads to a decrease in CD14 binding and decreased activation of immune cells (70, 71). Interestingly, HC1 also moderately increased TNF-α levels after 24 h, while IL-6 levels remained unaffected. A similar pro-inflammatory response has also been reported for the human AMP LL-37 (72).

### Salt sensitivity decreases LPS binding and challenges AMP development

One of the challenges in the clinical development of AMPs is their frequent loss of activity in physiological salt conditions (73). The increase in positively charged ions decreases the electrostatic interactions of the cationic AMPs with the anionic phosphate groups of the bacterial membranes, due to fewer available binding sites for the peptides (73 – 75). In our study, we also noted salt sensitivity for both HC1 and HC10. Although the AMPs were only minorly affected by high NaCl concentrations, the presence of divalent cations such as Ca^2+^ strongly decreased their antimicrobial activity. The BC assay confirmed that in physiological concentrations of CaCl_2_, the LPS-AMP binding was highly impacted. This indicates that LPS serves as an important anchor for the AMPs upon contact with the microbial membrane and is a second indication that the LPS-AMP binding is involved in their antimicrobial activity mechanism (75). To overcome this salt sensitivity, many structural modifications have been proposed. Amino acids such as histidine and tryptophan can be replaced by bulkier residues such as β-naphthylalanine (76, 77). This can lead to a deeper insertion of the AMP into the microbial membrane, protecting it partially from cation competition (76). Introducing lipophilic tags, including cholesterol or vitamin E, at the C-terminus or N-terminus of the peptide has also been proven successful in past research (20). Apart from structural modification, designing innovative formulations for AMP delivery can also increase salt resistance. Nanomedicine-based formulations such as liposome encapsulation and polymer-peptide conjugation are possible strategies to protect AMPs from salt interactions before they reach their target location (78, 79). For polymyxin antibiotics, these formulations have been proposed for inhalation therapy, shielding the peptide from the challenging lung environment (79). For HC1 and HC10, this could also be a valid strategy to work toward usable antibiotics against *P. aeruginosa* lung infections. Overall, despite promising properties such as strong antimicrobial activity and endotoxin-neutralizing properties, it is clear that both HC1 and HC10 will need optimization before any further routes to clinical applications can be considered.

## SUPPLEMENTAL MATERIAL

Supplemental figures msphere.00454-23-s0001.docx Figures S1 and S2.

ASM does not own the copyrights to Supplemental Material that may be linked to, or accessed through, an article. The authors have granted ASM a non-exclusive, world-wide license to publish the Supplemental Material files. Please contact the corresponding author directly for reuse.
