# Supplementary material for: In-depth biological characterization of two black soldier fly anti-Pseudomonas peptides reveals LPS-binding and immunomodulating effects
Source: mSphere. 2023 Oct 6;8(5):e00454-23. doi: 10.1128/msphere.00454-23 (PMC10597467; doi:10.1128/msphere.00454-23)
Supplement: Supplemental figures — Figures S1 and S2. [file msphere.00454-23-s0001.docx]

Supplementary data

S1 Effect of HC1 and HC10 on RAW264.7 cell viability


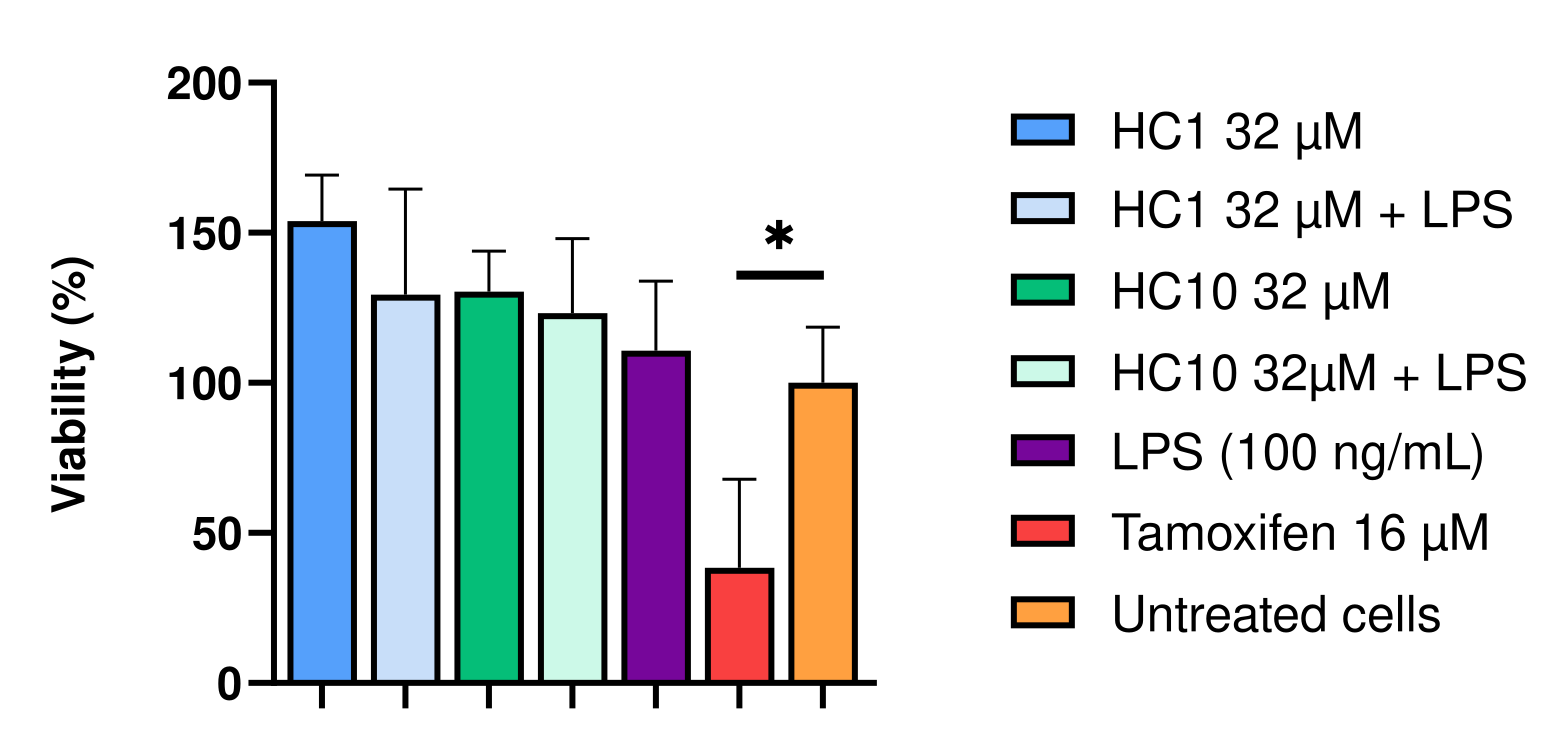


**S1 The effect of HC1, HC10 and/or LPS on the RAW264.7 viability.** The highest dose of HC1 and HC10 (32 µM) used in experiments was added to RAW264.7 macrophages, with or without the addition of LPS (100 ng/mL) to study the effect on cell viability after 48h of incubation. Data are represented as the mean + SD of the cell viability compared to a control of untreated cells. Tamoxifen was included as a positive control. Data were analyzed using a one-way ANOVA test.

S2 Effect of HC1 and HC10 on IL-1β and IL-12β production in LPS stimulated RAW264.7 cells


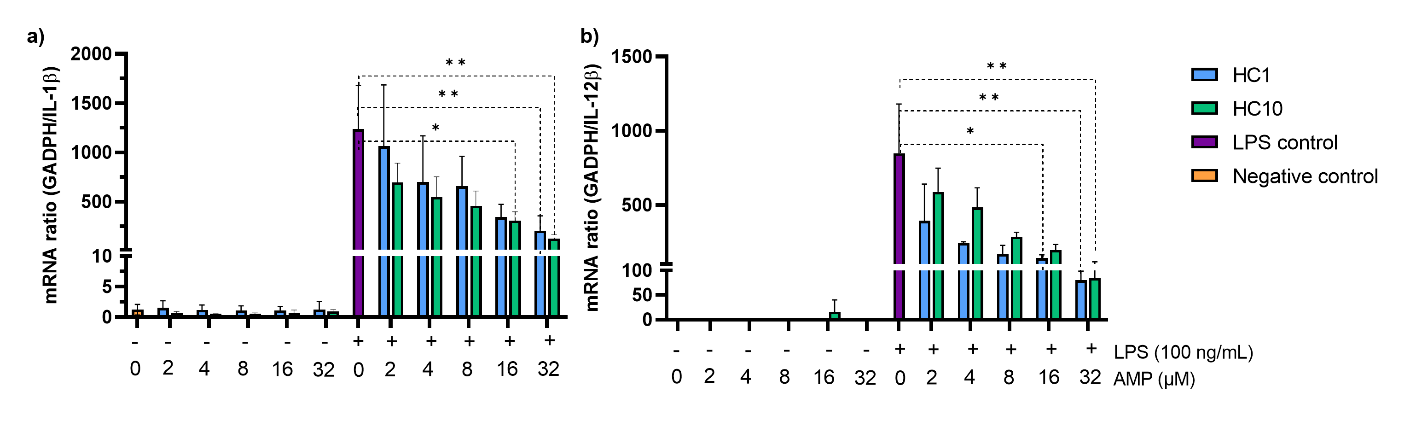


**S2 The effect of HC1 and HC10 on pro-inflammatory cytokine production in LPS-stimulated RAW264.7 cells.** a) The release of IL-1β by RAW264.7 macrophages after peptide monotreatment or LPS-AMP co-treatment for 24 hours was investigated using qPCR b) The release of IL-12β by RAW264.7 macrophages after peptide monotreatment or LPS-AMP co-treatment for 24 hours was investigated using qPCR. LPS treated cells (100 ng/mL) were included as positive controls. Data are represented as the mean + SD, and statistically analysed using a Kruskal-Wallis test. * = p ≤ 0.05, ** = p ≤ 0.01, *** = p ≤ 0.001, *** = p ≤ 0.0001. All experiments were carried out thrice.
